# Understanding Implementation of a Digital Self-Monitoring Intervention for Relapse Prevention in Psychosis: Protocol for a Mixed Method Process Evaluation

**DOI:** 10.2196/15634

**Published:** 2019-12-10

**Authors:** Stephanie Allan, Hamish Mcleod, Simon Bradstreet, Sara Beedie, Bethany Moir, John Gleeson, John Farhall, Emma Morton, Andrew Gumley

**Affiliations:** 1 Mental Health & Wellbeing, University of Glasgow Glasgow United Kingdom; 2 School of Behavioural and Health Sciences, Australian Catholic University Melbourne Australia; 3 Department of Psychology and Counselling, La Trobe University Melbourne Australia

**Keywords:** telemedicine, schizophrenia, implementation science

## Abstract

**Background:**

Relapse is common in people who experience psychosis and is associated with many negative consequences, both societal and personal. People who relapse often exhibit changes (early warning signs [EWS]) in the period before relapse. Successful identification of EWS offers an opportunity for relapse prevention. However, several known barriers impede the use of EWS monitoring approaches. Early signs Monitoring to Prevent relapse in psychosis and prOmote Well-being, Engagement, and Recovery (EMPOWER) is a complex digital intervention that uses a mobile app to enhance the detection and management of self-reported changes in well-being. This is currently being tested in a pilot cluster randomized controlled trial. As digital interventions have not been widely used in relapse prevention, little is known about their implementation. Process evaluation studies run in parallel to clinical trials can provide valuable data on intervention feasibility.

**Objective:**

This study aims to transparently describe the protocol for the process evaluation element of the EMPOWER trial. We will focus on the development of a process evaluation framework sensitive to the worldview of service users, mental health staff, and carers; the aims of the process evaluation itself; the proposed studies to address these aims; and a plan for integration of results from separate process evaluation studies into one overall report.

**Methods:**

The overall process evaluation will utilize mixed methods across 6 substudies. Among them, 4 will use qualitative methodologies, 1 will use a mixed methods approach, and 1 will use quantitative methodologies.

**Results:**

The results of all studies will be triangulated into an overall analysis and interpretation of key implementation lessons. EMPOWER was funded in 2016, recruitment finished in January 2018. Data analysis is currently under way and the first results are expected to be submitted for publication in December 2019.

**Conclusions:**

The findings from this study will help identify implementation facilitators and barriers to EMPOWER. These insights will inform both upscaling decisions and optimization of a definitive trial.

**Trial Registration:**

ISRCTN Registry ISRCTN99559262; http://www.isrctn.com/ISRCTN99559262

**International Registered Report Identifier (IRRID):**

DERR1-10.2196/15634

## Introduction

### Background

Psychotic disorders are common [1], and schizophrenia is one of the top 15 leading causes of disability worldwide [2]. Relapse is common in schizophrenia, with up to 80% of people experiencing one 5 years after onset [3]. Relapse is associated with increased costs to mental health services, with 70% of the UK mental health care costs being for unplanned inpatient hospital care for relapses [4,5] and a similar picture reported in Australia [6]. Relapse is associated with unwanted outcomes such as reduced social functioning [7]. Relapse also reduces the quality of life of both people with psychosis and their carers [8]. More frequent hospitalizations because of relapse are associated with reductions in relationship quality between service users and staff [9]. Staff wanting to intervene during early relapse report that they often struggle to engage with service users who have become mistrustful of their services [10]. In summary, relapses are associated with high financial and human costs, so detecting and intervening promptly to prevent the negative consequences of relapse is a crucial goal for schizophrenia care [11].

Relapse is the culmination of a process of changes that commence days and sometimes weeks before psychosis symptoms reemerge or are exacerbated [12,13]. These early warning signs (EWS) include affective changes and incipient psychosis. Although a Cochrane review of interventions targeting recognition and management of EWS of relapse in schizophrenia found significant effects for reduced relapse and rehospitalization rates [14], trial quality was poor regarding randomization, concealment, and blinding. Therefore, these interventions need to be more rigorously evaluated using high-quality randomized controlled trial (RCT) methodologies. Until this happens, relapse prevention interventions based on EWS cannot be recommended for routine implementation within health services [14].

Further barriers to implementation of approaches focused on EWS include their uncertain diagnostic utility [13], which may result in unnecessary intervention from mental health staff (false positives). Furthermore, in mental health services, the delivery of treatment through scheduled and routine appointments can result in EWS being missed because these experiences may not coincide with scheduled visits, thus reducing the opportunity for detection during times of actual need [15]. Finally, service users can be apprehensive about telling staff how they feel because this could trigger unwanted interventions such as hospitalization [16], which may act as a barrier to help seeking. Fear of relapse is linked to service users having more traumatic experiences of psychosis and hospital admission and greater fear of symptoms such as voices and paranoia [17] and experiencing fear of relapse appears to be linked to actual relapse events [18].

Digital interventions may enhance relapse prevention through the prompt identification and communication of EWS of relapse. The use of and enthusiasm for digital interventions for psychosis is reasonably high in service users [19-21], and current evidence of digital interventions’ acceptability and adherence rates suggests that these approaches are feasible [22]. Therefore, multiple strands of evidence suggest that it is time to develop a digital intervention to enhance relapse prevention and to test using RCT methodology. Implementation research explores the transfer of interventions from clinical trials into general usage [23]. Although RCTs are considered to be the most rigorous way of evaluating effectiveness in the medical context by providing substantial rigor and strong internal validity; in contrast, external validity (ie, implementation outcomes such as whether the intervention will be utilized within routine clinical practice) is often compromised [24]. Therefore, RCT methodologies alone may not answer research questions about implementation.

### Early Signs Monitoring to Prevent Relapse in Psychosis and Promote Well-Being, Engagement, and Recovery Study

Early signs Monitoring to Prevent relapse in psychosis and prOmote Well-being, Engagement, and Recovery (EMPOWER; ISRCTN: 99559262) is a proof-of-concept, cluster randomized controlled trial (c-RCT) to establish the feasibility of conducting a definitive RCT comparing EMPOWER against treatment as usual. This aim will be addressed by establishing the parameters of the feasibility, acceptability, usability, safety, and outcome signals of an intervention as an adjunct to usual care that is deliverable in the UK and Australian community mental health service settings. The EMPOWER study has approvals from the West of Scotland Research Ethics Service (GN16MH271 Reference 16/WS/0225) and Melbourne Health Human Research Ethics Committee (HREC/15/MH/344). The specific aims of EMPOWER are as follows:

1. to enhance the recognition of EWS by service users and their carers,
2. to provide a stepped care pathway, that is either self-activated or in liaison with a community health care professional (and a carer if a person has one), and
3. to then trigger a relapse prevention strategy that can be stepped up to a whole team response to reduce the likelihood of psychotic relapse.

EMPOWER is a just-in-time adaptive intervention (JITAI) [25]. JITAI is a term used to describe an intervention design that aims to address the dynamically changing needs of individuals via the provision of the type or amount of support needed at the right time and only when needed [26]. The EMPOWER app is a key part component of the EMPOWER intervention; the app prompts people with psychosis to input data once a day (through pseudorandom mobile phone invitations) via a repeated sampling method known as ecological momentary assessment (EMA) [27]. There are 22 questions that correspond to 13 different domains (activity, anxiety, coping, delusions, fear of recurrence, feeling threatened, hope, mood, other people, precipitants—such as sleep, seeing things, self, and voices—with an optional additional personal item) described further in the main trial protocol.

During the first 4 weeks of app usage, a baseline is established, which enables the EMPOWER algorithm to calculate the magnitude of future changes to support decision making. Following the baseline period, EMPOWER has the potential to trigger a response (decision point, in JITAI taxonomy) every time a participant responds to an EMA prompt (or fails to respond to a prompt for several days). Data entered by the participant responding to an EMA prompt were analyzed by the algorithm, resulting in one of the following responses: (1) if the algorithm detected no overall change in well-being, a generic message is randomly generated; (2) if the algorithm detected a small change (defined as an increase of over 1 SD from baseline) when a message tailored to the specific domain breach was generated. For example, if 1 SD change in sleep was detected, then the message featured sleep content; and (3) if the algorithm detected a higher change (defined as a change of over 2 SD away from baseline over 3 days), then this results in a check-in prompt (which is described further in the main trial protocol).

The EMPOWER system also allowed participants to use the app to view periodic graphs of their reported data (raw EMA data) and keep a diary of how they are feeling and why (stored locally only). Peer support workers helped set up and individualize the app for users and facilitated information exchange through their own lived experience of mental health problems to augment the individualized self-management aspect of support available via the app. Service users could review their app data with peer support workers as a means of promoting curiosity and reflection on the patterns of well-being over time. Regular telephone contact from peer support workers for the duration of the study aimed to maintain participant motivation for continued engagement with the app. Peer support worker calls also provided an opportunity for routine troubleshooting of any technical issues that arose with the app and for the identification of any adverse effects from the intervention.

The EMPOWER study aimed to recruit up to 86 service users between participating community mental health services in Glasgow (the United Kingdom) and Melbourne (Australia) along with staff members and relatives or carers (if the participant wishes this) who support a service user. EMPOWER meets the definition of a complex intervention by the Medical Research Council (MRC) [28]: it has various components, is being tested across 2 international sites, and includes mental health staff and carers as participants in addition to service users.

Mental health service users’ perspectives about interventions are rated low in the evidence hierarchy, with RCT evidence (especially in systematic reviews) coming out on top [29]. However, even with strong RCT evidence, no relapse prediction system for schizophrenia will be useful if it is not able to be integrated into clinical care and used by clinicians and patients [30]. Furthermore, a recent proof-of-concept trial for a digital intervention in psychosis concluded that more research was needed to understand service users’ and other stakeholders' perspectives on digital health systems to maximize implementation [15]. The design of digital interventions for mental health problems such as psychosis could be optimized if interventions are both valued by staff and patients and, therefore, compatible for long-term use and meeting clinical and scientific standards [31]. Use of current RCT methodologies in understanding complex interventions falls short of comprehensively explaining interventions [32]—with qualitative research being recommended [33] to enhance understanding. The benefit of qualitative implementation research exploring user experiences is illustrated by another study that identified barriers and facilitators to implementation for a digital intervention for bipolar disorder [34], which would have been missed if the focus was only on predefined outcome measures using a standard RCT approach. Service users reported that they felt motivated to use the intervention because of their positive relationships with the research team delivering the intervention.

Process evaluations are studies that run alongside a clinical trial, earning them the nickname of *trial siblings* [35]*.* Process evaluations look into the different components of a complex intervention, how it is delivered, and what happens when people interact with an intervention [36]. Process evaluations can improve the validity and interpretation of outcomes, help refine the intervention, and provide necessary information to help inform upscaling decisions for digital interventions. Therefore, a process evaluation will help answer questions about implementation that the EMPOWER c-RCT alone cannot [24]. In a pilot study such as EMPOWER, process evaluators are usually interested in facilitators and barriers to implementation so that strategies to ensure quality implementation can be put in place in time for a definitive evaluation [37]. A process evaluation can also support the development of implementation theories [37] that provide conceptual tools for researchers to understand, describe, and explain key aspects of dynamic and emergent implementation processes observed during trials for mental health interventions [38-40], including digital interventions for schizophrenia [41].

A process evaluation with a key focus on the usage of qualitative methods can enhance the understanding of the implementation process during the EMPOWER trial and illuminate user perspectives on key implementation issues such as acceptability, feasibility, and deliverability. As highlighted within their literature review of process evaluation frameworks, Marr et al [36] express concern that there is a common assumption within process evaluation frameworks that the interaction with an intervention is experienced in much the same way by different stakeholders and across different settings. We argue that given the complex and multicomponent nature of the EMPOWER intervention, the targeting of service users, carers, and mental health staff within the intervention program theory, and the intervention being tested across 2 international sites, it is doubtful that a process evaluator could identify key evaluation domains utilizing a predefined framework. Therefore, it was considered necessary to develop a process evaluation framework suited to the needs of trialists who wish to make decisions about potential upscaling and to ensure better that the needs of service users, carers, and mental health staff are addressed.

### Early Signs Monitoring to Prevent Relapse in Psychosis and Promote Well-Being, Engagement, and Recovery Process Evaluation Aims

In no particular order of importance, we aim to use the process evaluation for the following:

1. To understand the feasibility process of recruitment into the EMPOWER c-RCT by mapping out barriers and facilitators, which may be useful learning for a future full-scale trial.
2. To use the data collected after the recruitment is completed to develop a deep understanding of the experiences of the diverse group of stakeholders involved in the EMPOWER c-RCT, including members of the research team. A particular focus will be on identifying barriers and facilitators for implementation, acceptability, and feasibility.
3. To develop an implementation theory to understand and explain important aspects of the implementation process during the trial, including the impact of context (including psychological changes) on observed implementation outcomes.

We will now describe how the process evaluation aimed to address these through the development of a process evaluation framework and several key studies.

## Methods

### Process Evaluation Paradigm and Design

The MRC framework for process evaluations [37] highlights the importance of integrating mixed methods results from process evaluations to better understand what is observed within clinical trials. An explicit epistemological stance is also recommended as a way of reconciling the paradigms of quantitative and qualitative approaches within a single process evaluation [42]. However, our literature review suggests that epistemological positions invoked within process evaluations are not always reported within published protocols. We present a brief description of how we arrived at our epistemological stance, and how this shaped methodological choices.

Conjunctive theorizing (aiming to create appropriately complex rather than simplified abstractions of organizational phenomena) [43] is a recommended approach within implementation research [44] because such an approach situates implementation as subject to multiple interacting influences. With this in mind, it was decided to approach our process evaluation by choosing a research paradigm that focuses on understanding implementation from multiple stakeholder viewpoints. Constructivism presents such a paradigm [45]. Constructivism, although commonly assumed to be associated with qualitative enquiry, is not necessarily aligned with any particular methodological stance [46], and therefore, it provided no prescriptive guidance for methods chosen within our process evaluation. However, adopting a constructivist paradigm was critical in thinking about how to best develop research questions and choose methods that would maximize the understanding of participant experiences and develop a theory for interpreting these. This approach has been successfully used by Maar et al [36]. They reported that their approach resulted in process evaluation data that were relevant to their stakeholders and allowed for emergent understandings of implementation throughout the trial.

### Designing the Early Signs Monitoring to Prevent Relapse in Psychosis and Promote Well-Being, Engagement, and Recovery Constructivist Approach to Process Evaluation

**Figure 1:**
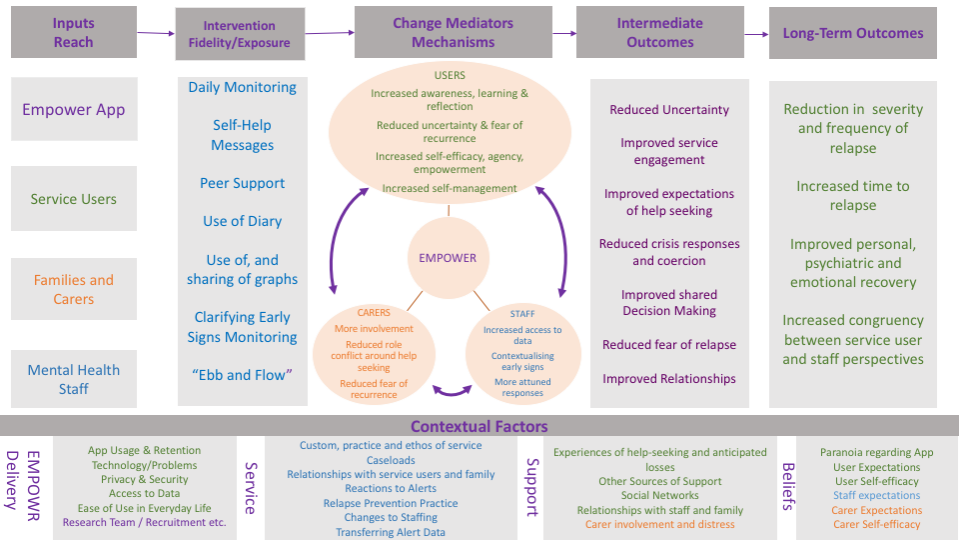
The logic model-based process evaluation framework for the Early Signs Monitoring to Prevent Relapse in Psychosis and Promote Well-Being, Engagement, and Recovery (EMPOWER) study.

Following the selection of an epistemological paradigm, the development of our process evaluation framework (Figure 1 was achieved through the following steps:

1. A process of mapping out the key EMPOWER components as listed in the trial protocol.
2. Analysis of key implementation themes constructed from formative qualitative work conducted before the trial involving 25 focus groups held with mental health staff, carers, and service users across international sites in both the United Kingdom and Australia [47].
3. A literature review of digital health evaluation issues, particularly those relevant to psychosis.
4. Choice and application of a process evaluation framework.
5. A final process of validity checking, where the proposed process evaluation framework developed from steps 1 to 3 was presented to researchers who had developed EMPOWER.

### Formative Qualitative Work

Following mapping out key EMPOWER components as described in the protocol (step 1), our formative qualitative work conducted in advance of the trial [47] was key to developing the process evaluation framework (step 2 of our process) and will be described briefly. The person-based approach to intervention evaluation [48] provided a useful guide for structuring the qualitative data as the process evaluation team developed the framework. Qualitative research is valued within the person-based approach because it allows exploration of participants’ understandings of factors or processes involved in intervention implementation. The formative qualitative work guided the process evaluators to develop process evaluation domains (based upon expected implementation facilitators and barriers) relevant to mental health staff, service users, and carers. For example, service user participants predicted that app user experience would be a key implementation factor, and therefore, the qualitative interview schedule (see Study 2A) had questions to explore this.

### Brief Literature Review of Psychosis-Specific Evaluation Issues

Our formative qualitative work also suggested both carers and service users (but especially service users) feel that they are in a disempowered position compared with staff within the current relapse management. Our literature review (step 4) identified that structural symbolic interactionism (a social theory) [49] had been used as a theoretical framework to understand power differences in interactions between mental health staff and service users in psychosis research within a constructivist paradigm [50]. When mental health staff believe someone diagnosed with a psychotic disorder is experiencing relapse, they prioritize their *risk management* role that has more positional power than role enactments focused on service user experiences [50]. In other words, service users reported feeling unable to influence decisions made by staff about treatment during this time and reported that their views of the situation were not valued.

Structural symbolic interactionism posits that individuals adopt positions that are recognized social categories (eg, being a carer). According to structural symbolic interactionism, a role is a set of expectations associated with a position, such as service user expecting a mental health professional to have a specific set of skills to manage relapse in psychosis [50]. At its heart, structural symbolic interactionism posits that people in their roles have agency when they interact both with each other and with interventions, but social structure always constrains interactions. For example, service user interactions are constrained by being in a relatively disempowered social role compared with the staff. Overall, although constructivism invites researchers to consider that all experiences are constructed, it falls short at explicitly considering how different people in their roles interact together and how existing power differentials might shape these interactions. Therefore, we used structural symbolic interactionism to enable us to think critically about power and ensuring the subjective views, and implementation experiences of relatively disempowered groups such as service users are valued in this process evaluation.

### Choice and Application of a Process Evaluation Framework

Our brief literature review also revealed a tension in process evaluation research, where research could be focused on implementation outcomes valued by mental health staff, service users, and carers [36] or be focused on addressing implementation outcomes valued by clinical researchers [37]. This was an important consideration because the overall aim of our process evaluation is to make an evidence-based comment on the acceptability, feasibility, and deliverability of the EMPOWER intervention. Although stakeholder implementation outcomes are important, they are not the whole story, and data also need to be suitable for researchers who work in clinical trials. Our attempt to address this tension within our constructivist paradigm is discussed next.

A logic model is a diagrammatic representation of an intervention, describing anticipated delivery mechanisms (eg, how resources will be applied to ensure implementation), intervention components (what is to be implemented), hypothesized mechanisms of impact (the mechanisms through which an intervention will work), and intended outcomes [51]. Logic models are recommended as a way of documenting the core functions of a process evaluation and providing a way to structure process evaluation findings. The logic model presented here (Figure 1) represents a process evaluation framework developed to be sensitive to the unique worldview of staff, service users, and carers. Choosing to incorporate the MRC process evaluation framework ensures that data generated during our process evaluation are valid for making accurate decisions about intervention implementation and improvement and also in contributing to the implementation research field more generally. In line with our constructivist paradigm, this pragmatic step reflected our view that trial researchers and staff are an active part of the enquiry and that process evaluation outcomes are not objective data but are shaped by researcher choices.

A lack of shared terminology within process evaluations can produce challenges when comparing process data from similar interventions across different trials [52,53]. This reduces the opportunity for inclusion of process data within systematic reviews. Utilizing the MRC process evaluation framework (enhanced by including the construct of *exposure* from the study by Matthews et al [54] to foreground the views of end users explicitly) provided the following taxonomy of key process evaluation terminology:

1. Reach: The extent to which the intervention reaches the target audience.
2. Fidelity: The extent to which the EMPOWER intervention is delivered as intended.
3. Exposure: The extent to which participants received and understood the different elements of the intervention.
4. Mechanisms of impact: The intermediate mechanisms through which an intervention creates an impact. This information is used to develop theories to understand why interventions reach implementation outcomes observed in trials.
5. Context: Factors external to the intervention that may influence its implementation or whether its mechanisms of impact act as intended.

Overall, our process evaluation framework builds upon the definition of context utilized within the MRC framework by considering what aspects of context are important for mental health staff, carers, service users, and researchers within the EMPOWER study and for valuing each group. Therefore, we hope that our process data will be specific enough to be relevant to the unique perspectives of our diverse stakeholders but general enough to allow for the inclusion of process characteristics within implementation evidence synthesis [55].

### Finalization of Process Evaluation Framework and Validity Checking

The validity of relationships posed within a logic model is reported to be strengthened through triangulation [56]. Therefore, the finalization of process evaluation (step 5) domains and the construction of the logic model (Figure 1) was facilitated through a discussion between the process evaluators and the research team. The final step was an iterative process involving critical feedback from members of the EMPOWER research team (including investigators and trial managers) working in both the United Kingdom and Australia. Ultimately, this step served as a final validity check to ensure that the proposed framework also made sense to the research team who had designed the intervention.

### Planned Process Evaluation Studies

The next subsection describes the planned process evaluation studies and their intended integration. As per MRC process evaluation guidance [37], all studies are based upon key areas of interest within our process evaluation framework (Figure 1), which is briefly described for each study in turn. The process evaluation studies were or will be carried out by a Doctor of Philosophy student (SA), a clinical psychology trainee (SBe), and a Master’s student (BM) who are semi-independent from the research team. SA and SBe are supervised by the chief investigator of the EMPOWER trial (AG). BM is supervised by SB and SA. For all studies, the process evaluators will be blind to any c-RCT outcome until it is published. Overall, all 6 studies inform each other by exploring implementation from the viewpoints of trial staff, mental health staff, service users, and carers.

### Study 1A: In-Depth Ethnographic Exploration of Recruitment

#### Background

Developing an understanding of the context of the recruitment process is important in understanding implementation feasibility [57]. Ethnography is recommended within process evaluation of complex interventions because this method enables process evaluators to understand process data within its social context and can produce internally valid data that can enhance the development of implementation theories [58]. Beyond standard ethnographic observations of how the researcher team carries out implementation processes, trial documents such as protocols and minutes of meetings are recommended as an essential source of ethnographic enquiry to understanding implementation more thoroughly [59].

#### Aim

The study aims to provide an account of the context in which recruitment to the trial occurred (Process Evaluation Aim 1).

#### Process Evaluation Framework

The process evaluation framework includes contextual factors.

#### Status

Data collection is complete, and analysis is ongoing.

#### Ethnography

A detailed analysis of minutes from meetings held in both the United Kingdom and Australia to provide a detailed account of recruitment concerning implementation feasibility and lessons for potential upscaling.

### Study 1B: Focus Group of Researcher Recruitment Experiences

#### Aim

The study aims to create an in-depth understanding of researcher insights about the recruitment process beyond what can be observed in ethnography (Process Evaluation Aim 1).

#### Process Evaluation Framework

The process evaluation framework includes contextual factors or EMPOWER delivery.

#### Focus Groups

After initial recruitment, the UK and Australian focus groups were run with the research assistants, trial manager, and chief investigator to enquire about their experiences of the recruitment process. A focus group schedule can be seen in Multimedia Appendix 1.

#### Status

Data collection is complete, and analysis is ongoing.

#### Analysis

Focus groups will be transcribed verbatim. Posttranscription, the focus group data will be analyzed inductively utilizing a thematic analysis approach [60]. All qualitative data will be stored in the latest version of NVivo, providing a transparent audit trail.

### Study 2A: Qualitative Interviews With Service Users, Carers, and Staff

#### Aim

The study aims to explore participants’ experiences of implementing and trialing the EMPOWER intervention, including their perceptions of any barriers and facilitators (Process Evaluation aim 2). Qualitative process data were collected through individually based in-depth interviews.

#### Process Evaluation Framework

The process evaluation framework includes all the factors.

#### Interviews

An interview guide was developed for each stakeholder group: mental health staff, carers, and service users. The service user interview schedule was developed to explore service user experiences of key components of the EMPOWER intervention (including *nondigital* areas such as interacting with peer support workers) as listed in the process evaluation framework. Mental health staff’s and carer’s interview schedules were developed to explore how these groups interacted with the intervention both directly and indirectly through interactions with a service user enrolled in the study. Furthermore, all interview schedules were designed to explore further anticipated mechanisms of change developed from formative qualitative work [47]—all schedules can be seen in Multimedia Appendices 2-4.

#### Participants

The participants include staff, service users, and carers in the United Kingdom and Australia.

#### Recruitment and Procedure

Within the United Kingdom, we purposively recruited a subsample of service users who provided their informed consent to participate in the EMPOWER study and who were randomized to the EMPOWER intervention arm. The purposive sampling strategy for approaching service user participants was developed from early-stage observations of the recruitment process. These early observations suggested that the following features might be relevant implementation factors: service user gender, service users inputting the same score every day (which would impact on the ability of the intervention to detect change), frequency of engagement with peer support workers, and whether a participant had experienced a relapse and an adverse event during intervention usage [61]. Therefore, we aimed to speak to participants who demonstrated a variety of the aforementioned characteristics to understand their experiences. We aimed to approach participants for interviews throughout the trial (following completion of baseline and during the 12-month follow-up period). The decision to collect qualitative interview data throughout the duration of the trial was to try and naturalistically capture the varied and evolving experiences of different participants over time.

Ethical approval for qualitative interview work with mental health staff, carers, and service users was received as part of an ethics amendment from West of Scotland Research Ethics Service (GN16MH271 Ref: 16/WS/0225) and Melbourne Health (HREC/17/MH/97 Ref: 2017.010). During the amendment application, it was decided by the ethics service that, because interviews with mental health staff and carers linked to a service user would involve them reflecting upon the service user’s experiences, mental health staff and carers will only be invited to participate in qualitative interviews if a service user provided their informed consent for this.

If a participating service user gave consent to the interview staff, we approached the mental health staff who had been involved in responding to ChIPs associated with changes in EWS or relapse episodes (as defined by the program theory) during their involvement in the study. If the service user provided consent to interview a carer, their carer was invited to participate soon after the service user was interviewed.

#### Status

Data collection has finished, and analysis not yet complete.

#### Analysis

Interviews will be transcribed verbatim. Posttranscription, the interview data will be analyzed inductively utilizing a thematic analysis approach [60].

### Study 2B: Qualitative Interviews with Early Signs Monitoring to Prevent Relapse in Psychosis and Promote Well-being, Engagement, and Recovery Trial Staff

#### Aim

The study aims to explore trial staff experiences of implementing key EMPOWER intervention components (peer support work and ChIPs), including their perceptions of any barriers and facilitators (Process Evaluation Aim 2). Qualitative process data were collected through individually based in-depth interviews.

#### Process Evaluation Framework

The process evaluation framework includes contextual factors or EMPOWER delivery.

#### Participants

The participants include peer support workers, trial staff involved in developing the peer support role within EMPOWER, and trial staff responsible for ChIPs.

#### Interviews

Interview schedules were developed for peer support workers and staff who are responsible for ChIPs. The interview schedule for peer support workers explores the delivery of peer support from the perspective of peer support workers by exploring their interactions with service users, which can include discussing EMPOWER app data. The interview schedule for trial staff involved in developing the peer support worker role explores their perceptions of how the peer support worker role has emerged from conception to delivery within the trial. Finally, the interview schedule for staff responsible for ChIPs explored the delivery of this intervention component from the perspective of the trial staff involved. All interview schedules are available in Multimedia Appendices 5-7

#### Recruitment and Procedure

All relevant trial staff members in both the United Kingdom and Australia were invited to take part in one-to-one interviews.

#### Status

Data collection has finished, and analysis is not yet complete.

#### Analysis

Interviews will be transcribed verbatim. Posttranscription, the interview data will be analyzed inductively utilizing a thematic analysis approach [60].

### Study 3: Development of Network Models

#### Background

The EMA data (daily ratings on a 1-7 Likert scale) generated through intervention usage was available to service users in its raw form via the graph function; service users could view their data and opt to share their data with others. However, the same data may reveal relationships between the well-being domains, which EMPOWER assesses. In network models, mental disorders such as schizophrenia are not conceptualized as common causes of symptoms but as conditions that arise from the interaction between symptoms [62]. A potential avenue of network research is the prediction of the course of mental distress from network characteristics of individuals. Network structure may demonstrate early warning signals*,* a term (distinct from EWS) describing temporal patterns of connectivity, which may indicate the upcoming onset of relapse for a specific individual [63]. Therefore, network models may present a useful means to quantify and understand the context of service user well-being during intervention usage and the relative influence of the 13 different well-being domains. In line with the EMPOWER program theory as defined in the protocol that will be published elsewhere, we are particularly interested in the fear of recurrence [18]. Little is known about such early warning signals in a relapse in psychosis, and it is hoped that exploring routine EMA data collected during the trial may provide an insight into the general phenomenology of well-being over time.

#### Aim

The study aims to better understand the context of service user well-being during intervention usage by building network models of psychosis during the stable, EWS, and clinical relapse phases—with the 3 states defined as per EMPOWER program theory (Process Evaluation Aims 2 and 3).

#### Process Evaluation Framework

The process evaluation framework includes change mechanisms or contextual factors.

#### Network Analysis

Exploratory network analysis will be performed using relevant packages on the most recent version of R.

#### Status

At the time of writing this paper, the data have not yet been analyzed in any form.

### Study 4: Exploratory Analysis of User Engagement

#### Background

Previous digital schizophrenia research studies use an EMA response rate of 33% for data to be considered reliable [64,65]. Although acknowledging that the criteria for determining EMA response feasibility varies in the literature [66], it is vital to determine what factors are associated with opportunities to maximize engagement. To the best of our knowledge, there are no guidelines for defining a required level of engagement with peer support. For example, a participant meeting a peer support worker 3 times was considered to be sufficient [67] but was not based on firm guidance. Therefore, there is a need to develop summary statistics about the levels of peer worker engagement.

#### Aim

The study aims to summarize and describe engagement with key components of the EMPOWER intervention and place these within a meaningful context (Process Evaluation Aim 2 and 3). Response to daily EMA prompts will be taken as a proxy for app usage. In addition, engagement with peer support will be defined from the number of actual peer support contacts compared with potential peer support worker contacts. Data will be analyzed retrospectively following completion of the trial.

#### Process Evaluation Framework

The process evaluation framework includes fidelity or change mechanisms.

#### Analysis

The analysis will include descriptive statistics of engagement levels (with both app and peer support) that will be triangulated with contact notes and qualitative process evaluation interviews.

#### Status

Usage data have been analyzed descriptively, but further analysis is not yet complete.

## Results

### Overview

At the time of writing this paper, no analysis is complete for any study. EMPOWER was funded in 2016, recruitment finished in January 2018. Data analysis is currently under way and the first results are expected to be submitted for publication in December 2019.

### Integration of Results

There is currently no consensus on what information is best for making decisions on whether an intervention is feasible for upscaling into a definitive trial [68]. Therefore, we recognized that data from the EMPOWER process evaluation could address a fundamental research question posed by Matthews et al: *Are identified barriers and challenges to implementation of the intervention planned for and surmountable?* [54]. In line with Matthews et al’s recommendations, the triangulated overall interpretation resulting from these studies will be presented as a strengths, weaknesses, opportunities, and threats (SWOT) analysis [69] that will list identified implementation barriers and challenges encountered during the EMPOWER intervention c-RCT, whether these were expected or unexpected, and if the process evaluation data suggest these are surmountable within an upscaled definitive clinical trial. This final result will be presented as an independent report to the relevant decision-making parties with recommendations (if relevant) for adaptations to the intervention.

## Discussion

### Principal Findings

This protocol describes 6 studies that utilize mixed methods to generate process evaluation data for the EMPOWER trial. These studies inform each other. The process evaluation data will be utilized to develop a SWOT analysis to more fully understand implementation within the EMPOWER pilot c-RCT through implementation outcomes constructed as being meaningful for mental health staff, carers, and service users. Ultimately, the findings from this process evaluation will provide evidence not available from other sources of evaluation within the trial to help inform upscaling decisions. Furthermore, the pilot c-RCT will allow the process evaluators to test the validity of the process evaluation framework by allowing for the emergence of unexpected outcomes within the implementation process. Any such implementation outcomes that deviate from the proposed framework will be used to restructure and refine the logic model to build a process evaluation framework that is more valid for understanding the actual implementation process.

Although the process evaluation framework was developed to be highly relevant to the process evaluation requirements for the EMPOWER study, this process evaluation may nonetheless provide data that are useful to other researchers. Theoretical understandings of how digital interventions create change are in their infancy; therefore, it is recommended that researchers prioritize qualitative methods [70] that foreground the discovery of how participants (in their own words) utilize interventions. Any potential benefit of digital interventions depends on users engaging with an intervention [71]. Engagement with digital interventions consists of 2 definitions: first, the extent to which an intervention is actually used (indicated by nonsubjective quantitative measures such as passively recording frequency of intervention usage), and second, as a subjective experience characterized by attention, interest, and affect (usually indicated through subjective measures such as questionnaires or interviews) [72]—concerningly, substantial heterogeneity in the use of measures has been noted [73]. Little is currently known about what aspects of a digital intervention are relevant for user engagement for a digital intervention for psychosis. This process evaluation will integrate nonsubjective measures (usage statistics) with subjective measures of engagement (through qualitative interviews) to develop a theory for understanding behavioral mechanisms underpinning engagement (or nonengagement) in people with psychosis.

To be suitable for fully informing behavioral change, theories need to capture individual differences and changes over time [74]. Most existing behavioral change theories lack utility for JITAIs because their static nature fails to capture the temporal dynamics of intervention usage over time [25]. Little is known about the subjective user experience of using JITAIs for psychosis. Therefore, the EMPOWER process evaluation provides an opportunity to develop an internally valid theory to better understand relationships between observable and objective measures of intervention usage with the subjective experiences of self-monitoring in people with psychosis. Such an understanding has broader implications for the management of psychosis and can inform the development of digital interventions for people with similar mental health problems, building on learning from previous qualitative work [75-80].

### Limitations

This research should be considered within its limitations. The formative qualitative work used to develop our framework included a large sample size for qualitative research. However, it is still not possible to make any claims about generalizability, and because this formative research was based on consultation and was not user led [81,82], its relevance to end users may be limited. Furthermore, there is a risk that important implementation outcomes were not uncovered through our prior qualitative work because of issues such as participants not feeling comfortable speaking within a focus group environment. Therefore, although the process evaluation framework appeared relevant to stakeholder needs constructed from focus group data, this is likely not a complete picture of actual stakeholder needs.

Participation within qualitative process evaluation interviews has been suggested [83] to represent a highly motivated group of service user participants who are not necessarily representative of the target population as a whole. Therefore, although discovering user insights in their own words is a key aspect of our constructive process evaluation approach, we may miss valuable user insight from this methodological choice. Furthermore, trial staff (who are members of the EMPOWER research team) may feel uncomfortable speaking freely within interviews because of the limited pool of participants, meaning that it may be possible to identify participants from quotes within qualitative data. A further significant limitation is that data collection ended for several studies before this protocol could be submitted for publication. However, formal data analysis was not initiated until the finalization of the protocol for publication.

### Conclusions

There are strengths to this study. By transparently stating our process evaluation development, aims, and proposed studies, we hope to contribute to good practice within this field [84] and share our learning. Publication of the protocol does not prohibit further process evaluation studies but ensures clarity that any such further study will be to explore unexpected consequences that were not anticipated within our predefined process evaluation framework. In line with recent recommendations to improve implementation research [53], the development of our constructivist process evaluation framework explicitly aimed to explore understandings between stakeholders and implementation science researchers.

## Abbreviations

c-RCT: cluster randomized controlled trial
EMA: ecological momentary assessment
EMPOWER: Early signs Monitoring to Prevent relapse in psychosis and prOmote Well-being, Engagement, and Recovery
EWS: early warning signs
JITAI: just-in-time adaptive intervention
MRC: Medical Research Council
RCT: randomized controlled trial
SWOT: strengths, weaknesses, opportunities, and threats

## Appendix

Multimedia Appendix 1 Focus group schedule for Study 1A.

Multimedia Appendix 2 Interview schedule for study 2A.

Multimedia Appendix 3 Interview Schedule for Process Evaluation work done on Study 2A.

Multimedia Appendix 4 Process evaluation interview schedule for service users.

Multimedia Appendix 5 Interview schedule for study 2B.

Multimedia Appendix 6 Interview schedule for Study 2B.

Multimedia Appendix 7 Interview Schedule for Study 2B.
