# Supplementary material for: Understanding Implementation of a Digital Self-Monitoring Intervention for Relapse Prevention in Psychosis: Protocol for a Mixed Method Process Evaluation
Source: JMIR Res Protoc. 2019 Dec 10;8(12):e15634. doi: 10.2196/15634 (PMC6930509; doi:10.2196/15634)
Supplement: Multimedia Appendix 1 [file resprot_v8i12e15634_app1.docx]

**Study 1A Recruitment Focus Group**

**Main Research Question**

What happened during recruitment, how might this help inform learning for a full trial?

**General Conversation Starters**

How do you think recruitment has gone overall?

What do you think have been the main difficulties in recruitment?

- Can you tell me a bit more about that?
- What has gotten in the way?
- What do you think has caused these difficulties? Why do you think they have happened?
- How have you negotiated these difficulties?

What have been the main strengths in recruitment?

- Can you tell me a bit more about that?
- What do you think has led to these strengths?
- What has helped?

If you could start again, how would you approach recruitment?

- What learning could be taken from these experiences?
