# Supplementary material for: Understanding Implementation of a Digital Self-Monitoring Intervention for Relapse Prevention in Psychosis: Protocol for a Mixed Method Process Evaluation
Source: JMIR Res Protoc. 2019 Dec 10;8(12):e15634. doi: 10.2196/15634 (PMC6930509; doi:10.2196/15634)
Supplement: Multimedia Appendix 2 [file resprot_v8i12e15634_app2.docx]

**EMPOWER KEY CLINICIAN INTERVIEW TOPIC GUIDE Study 2A**

**CONDUCTING THE INTERVIEW**

The following should be used as a guide only. Possible prompt questions are suggested that may be used where necessary to explore each of the topic areas is further detail.

The interview will comprise 3 main areas:

1. **Context**: The aim of this section is to gain insight into the context in which the episode occurred.

2. **The relapse episode**: The aim of this section is to hear the staff member’s experience of the relapse episode, their response within it and the role of the EMPOWER app.

3. **Reflection:** The aim of this section is to provide space for the participant to further reflect on the event in hindsight. The impact of the episode on subsequent care or the therapeutic relationship could be discussed. This section should also help the participant bring their focus back to the present moment and more neutral or positive topic, particularly if the interview has involved distressing emotions or topics.

**[P:** EMPOWER service user participant]

**PRE-INTERVIEW**

**Prior to commencing the interview, the research should ensure the following have been discussed with the participant:**

- Purpose of the research project
- Confidentiality
- Reminder of option to decline or withdraw participation at any time
- Any questions
- Ensure signed consent is completed locally

**INTRODUCTION**

“Thank you very much for agreeing to talk with me today. You know that I am interested in hearing about your experience of working with P during their recent episode of early warning signs/relapse. Part of the reason that we are conducting this research is that we know there can be a lot of challenges in providing care for people around times of relapse. I would encourage you to try to be as honest as possible.

We can start off by talking a little about the context of the episode; your relationship with P and what was going around the time of the relapse. I would then like to hear about your experience of working with P during the episode in as much detail as possible. I am interested in hearing about the details of what happened, but also your own thoughts and feelings in the situation and what factors influenced how you worked with P over that time. At the end we can spend some time reflecting back on the situation and whether you would do anything differently now.

I have some possible questions written here but I would like to talk as little as possible to allow you to tell your story.

How does that sound to you? Do you have any questions before we start?

|  | POSSIBLE PROMPTS |
| --- | --- |
| Context | - Generally speaking, what is your role when a person experiences EWS or relapse of psychosis? - Could you give me some context about your work with P?   - How long have you known them?   - What is their engagement like with you?   How would you describe your therapeutic relationship with P? With their family? |
| Relapse episode:    Staff **thoughts, feelings and behaviours** during the episode | - Can you talk me through the details of what happened. - What did you do? - What was going through your mind at the time? - How were you feeling? |
| Relapse episode:  **Systemic factors** implicated in staff experience of the episode and the provision of care | - What supports did you have? - Are there any expectations on you as a nurse? As part of the team? As part of the organisation? - What else was going on at that time? |
| Relapse episode:  R**elational context** for help seeking and service provision, including the staff/service user relationship and staff perceptions of the service user’s experience | - - - You have told me XX about your relationship with P/their engagement with you, how do you think that influenced your response on this occasion?     - How did P see the situation?     - What were P’s hopes/expectations/fears?     - What was P feeling?     - Were you aware of that at the time? How did that shape your response? |
| Relapse episode:  Role and impact of the **EMPOWER app** in the episode | - P had access to the EMPOWER app. Was it useful to you in this situation? Or to P? In what way? - Did you discuss/use the data? - Did the use of EMPOWER change or shape your interaction? - How did EMPOWER impact on you? On P? The relationship? - How did it affect your clinical decision making? - Could you see yourself using it in the long term? Why? (Strengths/ Difficulties) |
| Reflection | - How has your relationship with P been since? Has it changed at all? - Looking back now, would you do anything differently? - Did you learn anything from the experience? |
