# Supplementary material for: Understanding Implementation of a Digital Self-Monitoring Intervention for Relapse Prevention in Psychosis: Protocol for a Mixed Method Process Evaluation
Source: JMIR Res Protoc. 2019 Dec 10;8(12):e15634. doi: 10.2196/15634 (PMC6930509; doi:10.2196/15634)
Supplement: Multimedia Appendix 3 [file resprot_v8i12e15634_app3.docx]

## EMPOWER Carer Process Evaluation Study 2A

## Interview Schedule – Carers

## Version 1.0 21/01/2019

| **PRE-INTERVIEW**  **Prior to commencing the interview, the researcher based in Australia should ensure the following have been discussed with the participant:**   - Purpose of the research project - Confidentiality - Reminder of option to decline or withdraw participation at any time - Any questions - Ensure signed consent is completed, signed and retained   **Setting Up:**   1. Consent to be taken locally and then contact details given so researcher based in Glasgow can speak to the researcher 2. Introduction chat – see page 2. 3. Conduct interview   **Notes:**   - Greeting. - Verbal introduction to the aims of conducting process evaluation and asking carers/supporters what they think in their own words. Explain aim is for researcher to speak as little as possible and to listen to what they have to say. - Expected timings - Verbal explanation about recording device and confidentiality. - Any questions?   The interview schedule uses person you care for etc. throughout, but during interview the name of the person enrolled in the study will be utilized. “how has [named person] found X” |
| --- |

| **What** | **Questions** | **Prompts** | **Notes** | |
| --- | --- | --- | --- | --- |
| **Introduction** | How have you found the experience of being a carer in the EMPOWER study generally? | Have you filled in questionnaires with researchers etc?  How was that experience? Can you please tell me a bit more about that? |  | |
|  | Has the person you support mentioned accessing peer support, triage nurse, messages, daily-monitoring etc to yourself? | If so, in what ways? | I’d now like to ask some questions about your impressions of how the person you support has engaged with EMPOWER. As you know, EMPOWER also involves support from a triage nurse and peer support workers as well as access to messages etc. Would it be ok to ask some questions about these different parts? | |
|  | Do you feel you have been generally involved in EMPOWER as a carer/supporter? | If yes, in what ways. If not, why not. |  | |
| **Exposure** | If participant mentions that the person they care for/support has accessed key intervention components. | Use themes generated in response to “in what ways?” Ask what carer themselves thinks about key intervention components in addition to their impression on the thoughts and feelings of the person they support.  If carer states the person they care for has not accessed key components, ask why they think this might be.  If carer is unaware of the thoughts of person they care for, ask what carer themselves thinks about different parts. Give brief description of key component if necessary. |  | |
| **Change Mechanisms** | Since your loved one has started using EMPOWER, have you noticed any changes in how they manage their own well-being. | If yes, ask if they think this is related to EMPOWER use? | I’d now like to ask some questions about any changes that you may have noticed since the person you support has started to use EMPOWER? |  |
|  | Following on from above, have you noticed any changes in your own wellbeing? | If yes, ask if they think this is related to EMPOWER use? |  |  |
|  | What do you think and feel about relapse? Do you think what you think and feel about relapse has changed since being involved EMPOWER? Have you noticed any changes in how you and the person you support think or speak about relapse? | General - Can you tell me a bit more about that? |  |  |
|  | If you noticed things were “starting to slip” for the person you support – how do you think you would cope? Would the person you support using EMPOWER factor into this in any way? | Such as sharing data, relapse being identified early etc.  General – “can you tell me more about that?” |  |  |
| **Reach** | Is there anything else you’d like to tell us about your experiences of being a carer/supporter in this study? | General – Can you tell me more about that? | Finally, I’d like to ask a final few questions about your experiences using EMPOWER. |  |
|  | Is there anything you’d recommend changing? Or anything that you particularly liked? | Can you tell me more about that? Why is that? Etc.  Are there any changes that might be specifically useful for carer involvement? |  |  |
