# Supplementary material for: Understanding Implementation of a Digital Self-Monitoring Intervention for Relapse Prevention in Psychosis: Protocol for a Mixed Method Process Evaluation
Source: JMIR Res Protoc. 2019 Dec 10;8(12):e15634. doi: 10.2196/15634 (PMC6930509; doi:10.2196/15634)
Supplement: Multimedia Appendix 4 [file resprot_v8i12e15634_app4.docx]

## Process Evaluation of EMPOWER Study 2A

## Interview Schedule – Service Users

## Version 1.0 21/01/2019

| **PRE-INTERVIEW**  **Prior to commencing the interview, the Australia based researcher should ensure the following have been discussed with the participant:**   - Purpose of the research project - Confidentiality - Reminder of option to decline or withdraw participation at any time - Any questions - Ensure signed consent is completed, signed and retained   **Setting Up:**   1. Consent to be taken locally in Australia and then contact details given so researcher based in Glasgow can speak to the participant 2. Introduction chat – see page 2. 3. Conduct interview   **Notes:**   - Greeting. - Verbal introduction to the aims of conducting process evaluation and asking what service users think in their own words. Explain aim is for researcher to speak as little as possible and to listen to what they have to say. - Expected timings - Verbal explanation about recording device and confidentiality. - Any questions? |
| --- |

| **What** | **Questions** | **Prompts** | **Notes** | |
| --- | --- | --- | --- | --- |
| **Introduction** | How have you found using EMPOWER generally? |  | - Greeting, - Verbal introduction to the aims of conducting process evaluation and asking participants what they think in their own words. - Expected timings - Verbal explanation about recording device and confidentiality. Researcher desire for two recorders to be used in case of failure will be explained. - Any questions? - Taking informed consent. | |
| **Fidelity** | Did you use the EMPOWER App to monitor your wellbeing? | How was the experience? How often do you tend to use it? Do you take breaks? |  | |
|  | Did you access the EMPOWER messages? | If yes, in what ways? How were they useful or not? |  | |
|  | Did you use the graph function? | If yes, in what ways? Who with? If not, what put you off? |  | |
|  | Did you use the Diary function? | If yes, in what ways? If not, what put you off? |  | |
| **Exposure** | If Participant says they accessed the EMPOWER messages in any way.   - What did you think about the EMPOWER messages? | Use themes generated in response to “in what ways?” as prompts. |  | |
|  | If Participant says they used the graphs in any way.   - What did you think about the graph function? Were the graphs perceived as helpful? | Use themes generated in response to “in what ways?” as prompts. |  | |
|  | If participant says they used the diary function in any way   - What did you think about the diary function? | Use themes generated in response to “in what ways?” as prompts. |  | |
|  | How did you find interacting with the peer support workers? | Have you encountered a peer support worker before? Was peer support helpful in your use of the App? Did you learn anything about managing well-being? | - Introduction: As you know, EMPOWER also involves support from a triage nurse and peer support workers. Would it be ok to ask some questions about how you found this? | |
|  | How did you find interacting with the triage nurse? |  |  | |
|  | What did you think about how the App was designed? | Did it run smoothly?  Was it easy to use?  What did you think about graphics? |  | |
| **Context** | How did interactions with peer support workers (including phone calls) fit into your daily life? | - How did telephone calls from peer support workers fit into your daily life? | Introduction: I’d now like to ask some questions about how different aspects of EMPOWER have fitted into your daily life |  |
|  | How did self-monitoring with the App fit into your daily life? | - How do you feel about daily self-monitoring in relation to your personal schedule? - What did you think about alert timing? - Were there any times when it was easier or harder to self-monitor? |  |  |
| **Change Mechanisms** | Since you have started using EMPOWER, have you noticed any changes in how you manage your own-wellbeing? | If yes, ask if they think this is related to EMPOWER use? | I’d now like to ask some questions about any changes that you may have noticed since starting to use EMPOWER? |  |
|  | What do you think and feel about relapse? Do you think what you think and feel about relapse has changed since using EMPOWER? | Can you tell me a bit more about that? |  |  |
|  | If you noticed things were “starting to slip” – how do you think you would cope? Would you use any aspects of EMPOWER? | Such as sharing data, being attuned to experiences etc. |  |  |
| **Reach** | Is there anything else you’d like to tell us about your use of EMPOWER? | General – “can you tell me more about that?” | Finally, I’d like to ask a final few questions about your experiences using EMPOWER. |  |
|  | Is there anything you’d recommend changing? Or anything that you particularly liked? | Can you tell me more about that? Why is that? Etc. |  |  |
