# Supplementary material for: Understanding Implementation of a Digital Self-Monitoring Intervention for Relapse Prevention in Psychosis: Protocol for a Mixed Method Process Evaluation
Source: JMIR Res Protoc. 2019 Dec 10;8(12):e15634. doi: 10.2196/15634 (PMC6930509; doi:10.2196/15634)
Supplement: Multimedia Appendix 5 [file resprot_v8i12e15634_app5.docx]

**Topic Guide for Role of Peer Support Workers Study 2B**

**PRE-INTERVIEW**

**Setting up**

- Interviewer to organise date, time, and location of interview with interviewee
- Introductory conversation (see below)
- Begin interview (set timer)

**Prior to commencing the interview, the researcher should ensure the following have been discussed with the participant:**

- Greeting
- Purpose of the research project – aims
- Purpose of the interview
  - Aims (PSW, role, practicalities, the consumer)
  - Ask participant what they think in their own words
  - Explain aim is for researcher to speak minimally and to listen to what participant has to say
- Confidentiality and recording devices (two used in case of failure of one)
- The inclusion of potentially sensitive topics
- Reminder of option to decline or withdraw participation at any time
- Reminder that interview should last no longer than 60 minutes
- Any questions

Finally, ensure that signed consent is completed and retained locally as well as further verbal consent following above discussion.

**INTERVIEW**

*Interviewees:* ***Peer Support Workers***

| Investigative theme | Possible questions | Further prompts | Notes |
| --- | --- | --- | --- |
| The consumer | Can you tell me a bit about your role in EMPOWER?  What are your relationships with the consumers like? And could you tell me how they vary?  How have these relationships changed over time?  If you were one of your consumers, would you find the PSW beneficial? | Why do you think that is? Are there notable differences regarding gender, age, socioeconomic status, cultural upbringing (if you know these things about consumers)?  E.g., consumers becoming more comfortable with you; what lead to these changes? Are they for the better or worse? Can you give me an example?  Why? What would you keep the same and what would you change? | I would like to ask you some questions about the service users from your perspective as a PSWer. Please answer as honestly as possible, and remember there are no wrong answers. |
| Practicalities | What do you think of your PSW role occurring through the telephone? Advantages/disadvantages?  What do you think your consumers think of this?  Have any of your consumers ever commented on this - whether they like it or not?  Has a consumer ever asked to meet in person?  What do you think of the amount of contact you have with your consumers?  (*If would change amount of contact*) to what?  What do you think the consumers think about amount of contact?  I understand the consumers vary in their level of engagement and some want less contact. What do you think contributes towards this difference – those who are engaged vs those who are not?  Do you think it would be beneficial to specifically match consumers to their PSWers? | Telephone counselling is a common practice.  Ask to elaborate why. If would change - to what? And why do you think this would be better?  How much contact do you have? Is this enough and why?  And why do you think this would be better?  Have they ever talked to you about this? What did they say?  What encourages vs discourages engagement with support? E.g.., gender, age, culture, first language?  Have you experienced any matching? And what do you think would they could be matched on? E.g., gender, others? | I would now like to ask you some more practical questions regarding your role as a PSWer. |
| Role | Before this project have you had any PSW role training or experience?  Have you had any other PSW roles before this one?  What do you believe a PSW role should entail?  Before beginning, what did you think this PSW role would entail? (In line or not in line with previous question)  Is this PSW role as you expected PSW to be or different?  Is your role ever counter to what you think PSW should be?  How has your role changed over time?  Do you believe you gain anything by being a PSWer in this study? - Supposed to be bidirectional support  (**Sensitive – warn**) What challenges do you face because of your role?  I understand you later became able to see the consumers’ app data. Did this have an impact on your relationship with them?  Do you believe there is a power imbalance between you and the consumer? I.e., you have access to their data, you have formal training  Is there anything else at all you would like to tell me? | If yes - when and what did the training involve? What do you think of the training? Good or bad? How could be improved/made clearer?  How many and where/when/with who?  In an ideal world - what should the consumer gain from the PSW? What should the PSWer gain? What negatives should be removed from it? Asking about values of PSW; mutuality and bidirectionality of support, less power imbalance for e.g.  Benefits and removal of negatives?  If have had previous PSW role - same or different? General benefits and weaknesses of your role to you and consumer (and project?)  Is your work in keeping with values of PSW as you understand them?  E.g., consumers becoming more comfortable with you; what lead to these changes? Are they for the better or worse? Can you give me an example?  E.g., work experience, paid employment, sense of capability/achievement, someone to talk to - do you get to talk yourself too?  E.g., potentially triggering, stress and responsibility, inability to provide enough support, frustration from role (e.g., not what thought), boundaries, accountability, power dynamics, NHS employed, MH worker despite PSWer, can see data  For better or worse? How has role changed over time?  If yes - how do you think this affects your relationship with the consumer (from both perspectives) and the consumers’ response to the treatment | Finally, I would like to talk to you about your role as a PSWer.  You may find this to be a sensitive question, and we recognise that due to the small number of PSWers there is a possibility you could be identifiable. Remember, you can share as much or as little as you like. You need not answer at all if you would prefer. |
