# Supplementary material for: Understanding Implementation of a Digital Self-Monitoring Intervention for Relapse Prevention in Psychosis: Protocol for a Mixed Method Process Evaluation
Source: JMIR Res Protoc. 2019 Dec 10;8(12):e15634. doi: 10.2196/15634 (PMC6930509; doi:10.2196/15634)
Supplement: Multimedia Appendix 6 [file resprot_v8i12e15634_app6.docx]

**Topic Guide for Role of Peer Support Workers Research (Study 2B)**

**PRE-INTERVIEW**

**Setting up**

- Interviewer to organise date, time, and location of interview with interviewee
- Introductory conversation (see below)
- Begin interview (set timer)

**Prior to commencing the interview, the researcher should ensure the following have been discussed with the participant:**

- Greeting
- Purpose of the research project – aims
- Purpose of the interview
  - Aims (PSW, role, practicalities, the consumer)
  - Ask participant what they think in their own words
  - Explain aim is for researcher to speak minimally and to listen to what participant has to say
- Confidentiality and recording devices (two used in case of failure of one)
- The inclusion of potentially sensitive topics
- Reminder of option to decline or withdraw participation at any time
- Reminder that interview should last no longer than 60 minutes
- Any questions

Finally, ensure that signed consent is completed and retained locally as well as further verbal consent following above discussion.

**INTERVIEW**

*Interviewees:* ***Key Informants***

| Investigative theme | Possible questions | Further prompts | Notes |
| --- | --- | --- | --- |
| Peer support work (PSW) | What is the purpose and desired outcomes of PSW generally?  Why were PSWers included in project?    Do PSWers role in this study agree or conflict with underpinning values of PSWers?  Do the PSWers provide added unique value to the consumers? Or could anyone do this?  Do you believe the PSWers gain anything by participating in this study as PSWers?  Do the PSWers face any challenges?  Do the PSWers receive adequate training? | Underpinning values  Why did you include PSWs in the EMPOWER study?  Intended added value; to the consumers and to the project; functionality - app  Elaborate - why if yes or no; functional vs supportive  Additionality  Supposed to be bidirectional support  E.g., potentially triggering, stress and responsibility, inability to provide enough support, frustration from role (e.g., not what thought), boundaries, accountability, power dynamics, health system employed, MH worker despite PSWer, can see data  Ongoing support with role and beyond (general life); above challenges | I would like to first ask you some questions about PSW generally. There are no right or wrong answers, I am just interested in your personal perspective.  Be conscious of sensitivity here |
| PSWers role | What do you believe PSW role *actually is*? I understand you are now encouraging they assist with self-management/recovery  Can PSW role be supportive and practical?  What was the intended role of PSWers?  Expectation of PSW knowledge and competencies in line with level of employment/role?  To what extent have PSWers adopted the role that was envisioned?  How have the PSWers roles changed over time?  Risk of “miracle workers” of PSW due to lived experiences  Are the intended outcomes of PSWers being achieved?*  Is there a power imbalance between the PSWers and consumers?  Is there anything else at all you would like to tell me? | Mental health (supportive) and/or digital help (practical)? How much of their role is each?  Is one more beneficial/unnecessary? Is one PSWer enough to provide both roles?  Intended outcomes?*  Do we have realistic expectations of PSWers? Reasonable to expect them to do this? Too hard or too easy? Potentially very complex and sensitive topics, needs of consumers; too big an ask? NHS Band 3  Have practices developed?  There are still no easy answers/solutions to psychosis; treatment success varies  Enough for validity and replicability of study? Enough to produce valid results and conclusions?  I.e., PSWers have access to consumers’ data, they have training, they are paid employees; supposed to be peers – equal relationship? But still, chose PSWers, additionality? | I would now like to move on to ask you more specifically about the PSWers in the EMPOWER intervention. Please answer as honestly as possible from your personal perspective as a key informant. |
