# Supplementary material for: Understanding Implementation of a Digital Self-Monitoring Intervention for Relapse Prevention in Psychosis: Protocol for a Mixed Method Process Evaluation
Source: JMIR Res Protoc. 2019 Dec 10;8(12):e15634. doi: 10.2196/15634 (PMC6930509; doi:10.2196/15634)
Supplement: Multimedia Appendix 7 [file resprot_v8i12e15634_app7.docx]

**EMPOWER TRIAL STAFF PERSPECTIVES INTERVIEW Study 2B**

**CONDUCTING THE INTERVIEW**

The following should be used as a guide only. Possible prompt questions are suggested that may be used where necessary to explore each of the topic areas is further detail.

The interview will comprise of two areas.

1. Description. The aim of this section is to enable the participant to describe their experiences of responding to Check in Prompts (CHiPs) in some detail including interactions with consumers/key-clinicians/carers and other members of the research team.
2. Reflections. The aim of this section is to create space for the trial team member to reflect upon the work of triaging and responding to CHiPs.

**Pre-Interview**

**Prior to commencing the interview, the researcher should ensure the following have been discussed with the participant:**

- Purpose of the research project
- Confidentiality
- Reminder of option to decline or withdraw participation at any time
- Any questions
- Ensure signed consent has been completed locally

**Interview**

**“**I’d like to start by asking you about your role in triaging CHiPs before moving on to any clinical response which may occur from them. Would that be ok?”

|  | POSSIBLE PROMPTS |
| --- | --- |
| Description - Context | - Generally speaking, what is your role in triaging a CHiP? - What information do you use in the triage process? Why do you think you make the decisions you do? - Is anyone else in the EMPOWER involved in triage? - What is their role? - What other tasks are associated with your role? - Has this role changed over time? - How much time does triaging take? How do you balance this with your other tasks? - Are there differences in how CHIPs are triaged for different participants? Can you give me an example?   “I’d now like to move on from triaging to asking about responses”   - - What do you do if triage suggests a CHiP needs to go further? Who do you contact? What is their role? What usually happens? Who else becomes involved? Such as key clinicians / carers. Can you give me an example?   - Are there participant differences? Can you give me an example?   - Have you encountered any “false positive” (defined as EMPOWER triage suggesting support is needed but consumer / key clinician indicating that it was not).   - What happened? |
| Reflections | “I’d now like to ask you more about what you have learned doing this role”   - Are there any challenges in triaging and/or responding to CHiPs? - What do you think leads to challenges? - How do you respond to challenges? - Are there any expectations on you as an individual in triaging or responding to CHiPs? As a trial team member? As part of wider organisation (such as a health board) - What do you think of the utility of EMPOWER data in alerting us to significant changes in wellbeing? What is your view of discontinued usage alerts? - How do you think consumers / key-clinicians / carers find the triage / response process? What makes you think that? - What do you think could improve your role in triaging or responding to alerts? - Are there any comments or suggestions you have for EMPOWER going forward? How about tailoring of check is to match consumer preferences (e.g. texting participants or alerting key workers?) |
